# Posterior thigh compartment syndrome secondary to proximal hamstring avulsion

**DOI:** 10.1016/j.ijscr.2024.110747

**Published:** 2024-12-16

**Authors:** Herijaona Manasse Rakotoherisoa, Thomas Daoulas, Manitr'Oliva Iarimanalina Ranaivoson, Marie Fernandez, Aadel Hamdadou, Nacer Debit

**Affiliations:** a CHU Joseph Ravoahangy Andrianavalona, Service de Chirurgie Orthopédique et Traumatologique, 101 Tananarive, Madagascar; b CHU Cavale Blanche, Service de Chirurgie Orthopédique et Traumatologique de Brest, Boulevard Tanguy Prigent, 29200 Brest, France; c CHI de Poissy - Saint-Germain-en-Laye, Service de Chirurgie Orthopédique et Traumatologique, 78300 Poissy, France

**Keywords:** Aponeurotomy, Hematoma, Proximal hamstring injury, Sciatic nerve, Thigh compartment syndrome

## Abstract

**Introduction:**

Thigh compartment syndrome is a rare clinical entity that requires urgent medical and surgical management due to its potentially severe medical and legal consequences. Although it typically affects a classic patient demographic—most often a young adult following a traffic accident.

**Presentation of case:**

This case is unique in that it stems from a compressive hematoma secondary to an avulsion of the proximal hamstring tendon.

**Discussion:**

Thigh compartment syndrome can be challenging to diagnose due to its sometimes-misleading clinical presentation. Hamstring rupture is a very rare etiology and may lead to a delay in diagnosis and management, which can have serious consequences.

**Conclusion:**

We report a rare case of thigh compartment syndrome secondary to hamstring rupture.

## Highlights

• Rare case of thigh compartment syndrome due to hamstring tendon avulsion
• Early diagnosis and fasciotomy crucial to prevent severe complications
• MRI useful for hamstring injury evaluation when emergency imaging is feasible
• Delay in treatment may lead to sensory deficits despite complete motor recovery.
• Timely interdisciplinary care essential for optimal patient outcomes

## 1 Introduction

Thigh compartment syndrome is a rare condition associated with high long-term morbidity and mortality [[1], [2], [3]]. It is a surgical emergency that occurs when the pressure within the fascial compartment of the thigh exceeds perfusion pressure, leading to cellular anoxia of the various structures within the thigh compartment [2]. It is even more unusual when secondary to a proximal hamstring rupture [4,5]. The typical mechanism is a sudden motion involving hip flexion and knee extension, which maximally stretches the hamstrings; avulsion occurs when they contract eccentrically [[5], [6], [7]].

Early surgical treatment is essential, and bone reinsertion of the avulsed tendon at the ischial tuberosity is preferable immediately following fasciotomy and assessment of the muscle-tendon injury [8].

## 2 Presentation of case

We report the case of a 60-year-old male, BMI 32, with a history of hypertension and type II diabetes. The patient was not taking anticoagulants or antiplatelet agents. Following a 10 kg load-bearing event with his hip in external rotation and flexion and his knee in full extension, the patient described a sharp, excruciating snapping sensation at the back of the thigh, followed by complete functional impairment. Written informed consent was obtained from the patient for publication and any accompanying images. A copy of the written consent is available for review by the Editor-in-Chief of this journal on request. The work has been reported in line with the SCARE criteria.

The patient presented with a painful swelling, VAS 10/10, firm to palpation, located on the anterointernal and posterior thigh. Initial clinical examination revealed hypoesthesia throughout the lower limb without motor deficit, and distal pulses were present.

A contrast-enhanced CT scan showed a 30 cm hematoma in the posterior thigh compartment, between the biceps femoris and semitendinosus, along with a proximal hamstring tendon avulsion.

The combination of factors—pain resistant to opioid analgesics, preserved distal pulses, excessive skin tension, and an identified cause on CT imaging—led to the diagnosis of thigh compartment syndrome. To optimize surgical management, despite the theoretical delay in treatment, we decided to proceed with surgical intervention without performing additional examinations.

An emergency surgery was performed 24 h after the initial trauma, involving decompressive aponeurotomy via an anterointernal approach, with hematoma evacuation and loose closure for decompression of adductor compartment and posterior compartment.

An anteromedial incision typically allows the vastus lateralis to be retracted medially to expose the lateral intermuscular septum and access the posterior compartment. However, this incision does not provide access to the adductor compartment, which exhibited minimal clinical symptoms. It should be noted that we did not perform the anteromedial incision because the anterior compartment, including the quadriceps and sartorius muscles, was completely asymptomatic.

Postoperative outcomes included persistent sensory deficits in the L4-L5 dermatome. MRI conducted due to sensory symptoms revealed a 10 × 8 cm hematoma between the gluteus maximus and external obturator, secondary to proximal hamstring avulsion compressing the sciatic nerve.

**Fig. 1:**
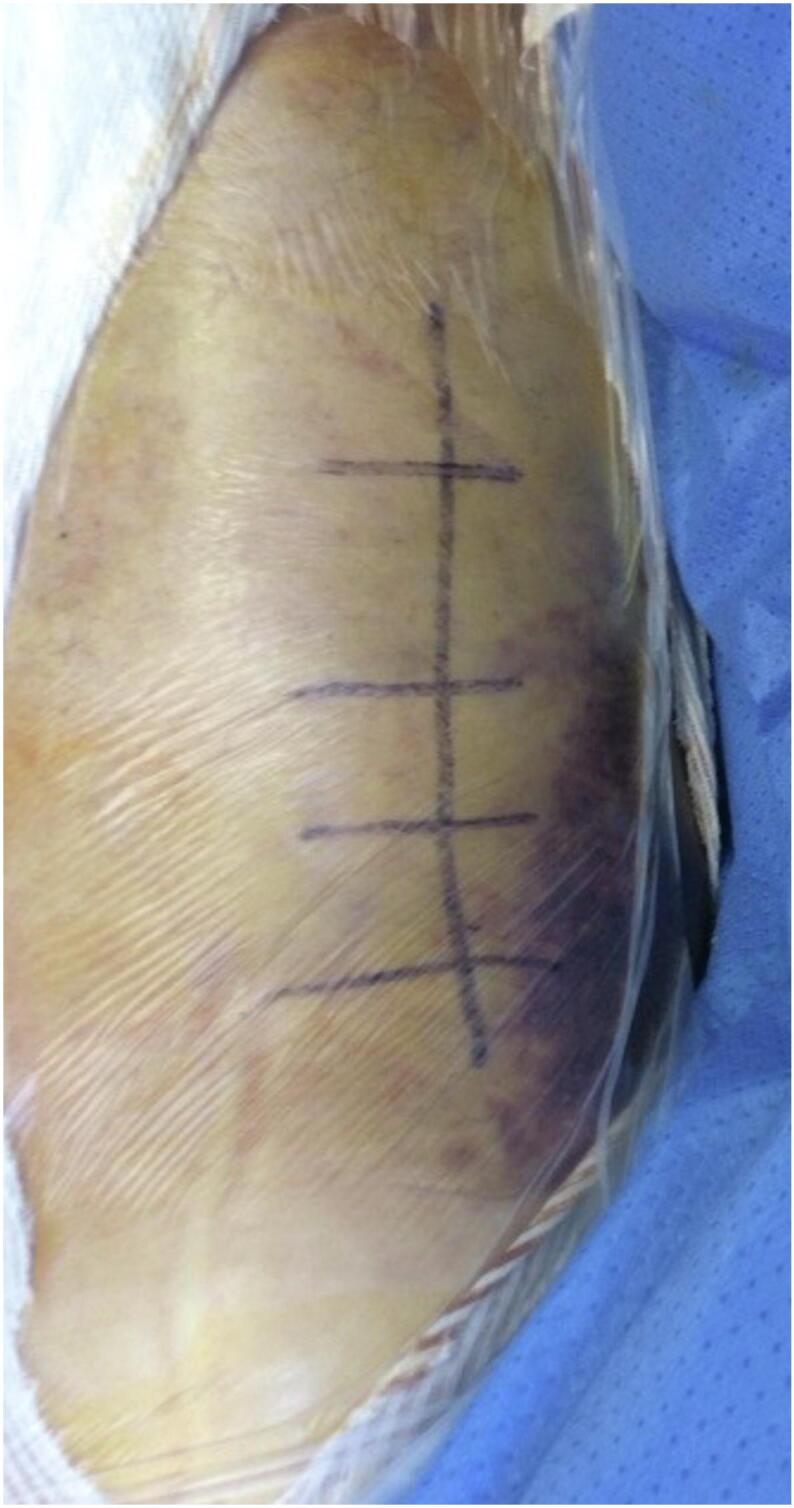
Posterior approach to the right thigh for decompression fasciotomy.

A second surgical intervention was performed seven days after the first decompression aponeurotomy, beginning with a posterior approach decompression of the proximal two-thirds of the posterior thigh compartment: a longitudinal skin incision was made on the midline of the posterior thigh, extending from below the gluteal fold to the middle third of the thigh (Fig. 1).

**Fig. 2:**
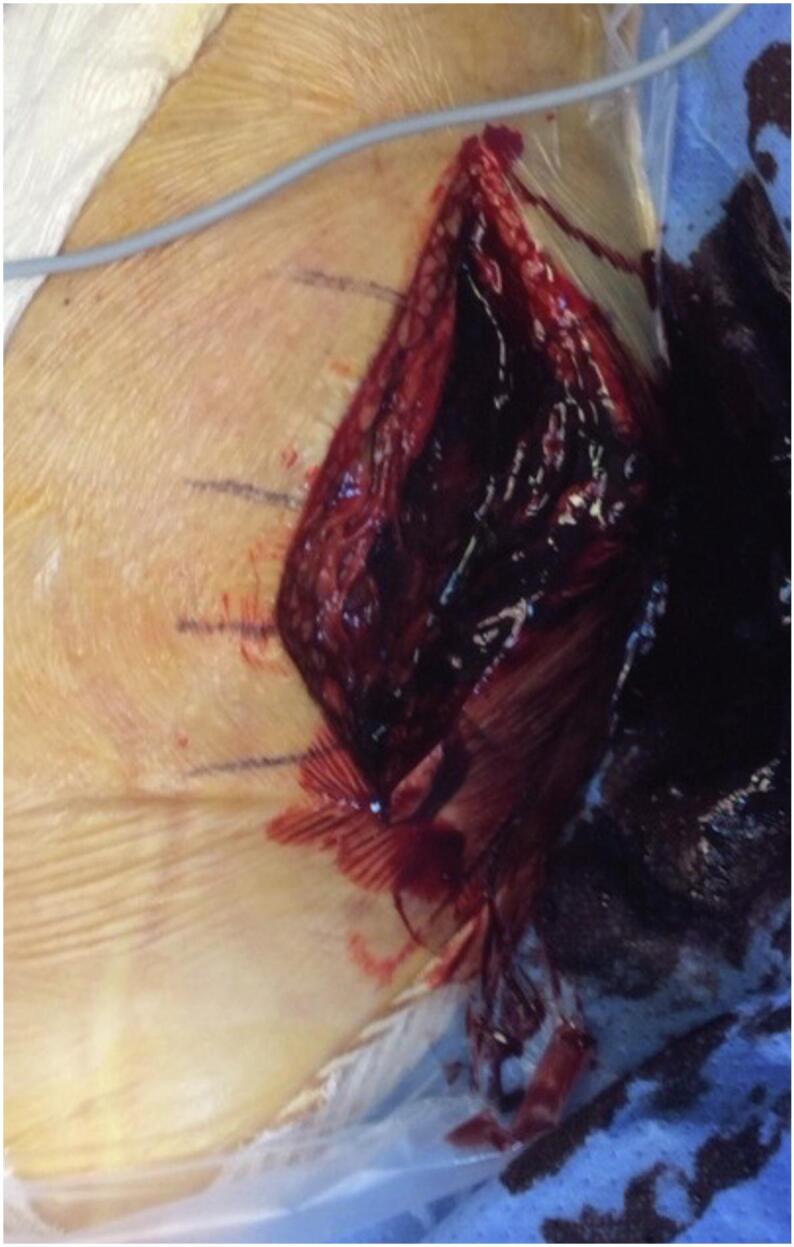
Evacuation of the hematoma compressing the sciatic nerve.

**Fig. 3:**
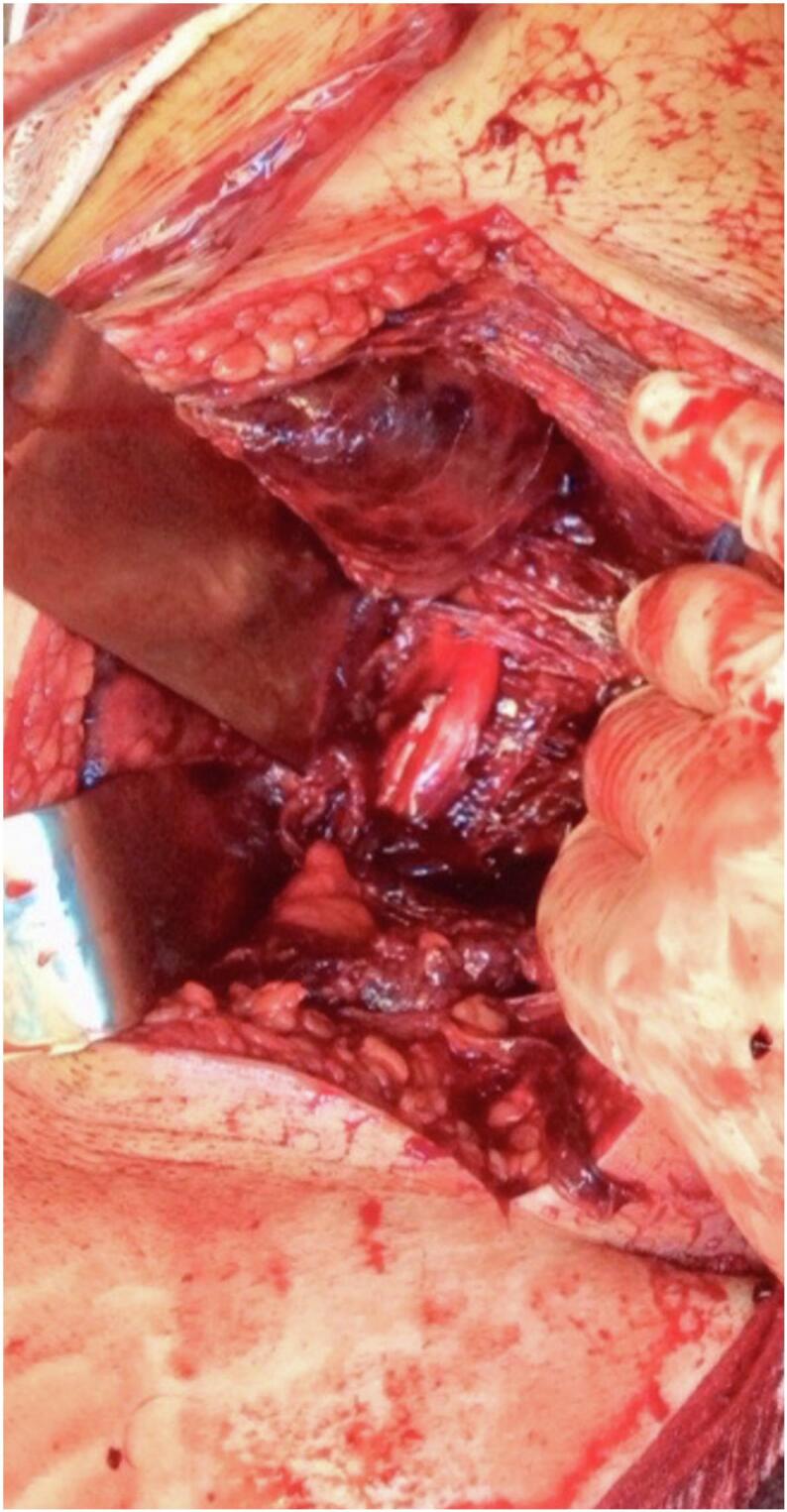
Integrity check and neurolysis of the sciatic nerve.

The fascia was incised along the skin incision while preserving the sciatic nerve's cutaneous branch, followed by hematoma evacuation (Fig. 2) and sciatic nerve neurolysis (Fig. 3) with transosseous reinsertion of the hamstring tendons using an anchor. Finally, the initial anteromedial incision was closed.

Three weeks postoperatively, the patient showed favorable progress with decreased pain and gradual sensory recovery. At a one-year follow-up, there was satisfactory progress with complete motor recovery in all thigh and leg muscles, although some dysesthesia persisted in the L4-L5 dermatome on the right foot.

## 3 Discussion

Acute compartment syndrome is a medical-surgical emergency caused by elevated pressure within a closed compartment, exceeding capillary perfusion pressure, leading to cellular anoxia and compromised tissue viability. Without timely decompressive fasciotomy, it can result in irreversible tissue ischemia and necrosis [9]. The mechanism is most often post-traumatic due to vascular injury or fracture-related injuries [10] and, as in our case and a few recent studies, proximal hamstring rupture [11]. The hamstring complex consists of three muscles working in synergy for hip extension and knee flexion. Except for the short head of the biceps femoris, the other hamstring muscles cross both the knee and hip joints, making them more susceptible to proximal injury [6]. With this case, we aim to identify ways to achieve early diagnosis and implement appropriate emergency treatment. Rodriguez et al. demonstrated in their 2020 study that delays in treatment of over 24 h via fasciotomy can result in severe motor or sensory deficits, often with irreversible ischemic damage and associated legal consequences, emphasizing the importance of early diagnosis [2,9,10]. In our case, despite delayed surgical management, the patient did not develop major sequelae at the 10-month follow-up; however, residual sensory deficit may be associated with the delay in initial treatment. In our case, surgical treatment was initiated 24 h after the trauma but likely not long after the onset of thigh compartment syndrome. Indeed, after six hours, surgical treatment is generally no longer indicated and may even be contraindicated due to the significantly increased risk of postoperative infection. We believe that this thigh compartment syndrome developed insidiously due to an extensive compressive hematoma rather than immediately following the trauma.

Furthermore, given the limited data available in the literature, it is plausible to hypothesize that the thigh, with its compartments being significantly larger compared to those of the leg or forearm, may better tolerate extensive processes before progressing to compartment syndrome. This could explain the delay between the initial trauma and the onset of symptoms associated with compartment syndrome.

This case does not exhibit the typical patient profile for thigh compartment syndrome, which most commonly affects young individuals, patients with coagulation disorders, or those on long-term anticoagulant therapy [9,11,12]. Unlike leg compartment syndrome, thigh compartment syndrome is rare and more challenging to diagnose due to its clinical presentation and clinicians' lack of experience, especially in unconscious patients following polytrauma [2]. Diagnosis can be confirmed by intramuscular pressure measurements, though they should not delay surgical intervention [13].

Some studies in the literature recommend an emergency vascular imaging workup, such as an angio-CT scan, to support the diagnosis [10]. Initial diagnosis is even more challenging due to less marked symptomatology compared to compartment syndromes of other segments like the leg or forearm, further compounded by clinicians' limited experience due to its rarity [12]. Outside emergency situations and depending on equipment availability, MRI is the preferred examination to precisely evaluate pathology associated with hamstring tendon avulsions. This examination was not indicated in our case due to the urgent nature of the symptoms [7].

The work has been reported in line with the SCARE criteria [14].

## 4 Conclusion

Thigh compartment syndrome secondary to proximal hamstring tendon avulsion is an extremely rare clinical entity with potentially catastrophic consequences if treatment is delayed. It requires close and rapid collaboration among healthcare providers.

## CRediT authorship contribution statement

HMR: Conceptualization; Data curation.
TD: Formal analysis; Funding acquisition.
MF: Investigation; Methodology.
MOIR: Project administration; Resources.
AH: Supervision; Validation; Visualization.
ND: Roles/Writing - original draft.

## Consent

Written informed consent was obtained from the patient for publication and any accompanying images. A copy of the written consent is available for review by the Editor-in-Chief of this journal on request.

## Ethical approval

The local ethics committee approved the collection and publication of these data.

## Guarantor

Thomas Daoulas.

## Declaration of Generative AI and AI-assisted technologies in the writing process

All authors certify that they have not used AI in the writing, data collection and design of this article.

## Funding

None.

## Declaration of competing interest

The authors declare no conflict of interest.

## References

[1] G. Verwiebe E, M. Kanlic E, Saller J, et al. Thigh compartment syndrome, presentation and complications. Bosn J of Basic Med. Sci. 2009; 9: S28–S33. 10.17305/bjbms.2009.2751 PMC5655168 19912116

[2] Knab L.M., Abuzeid A., Rodriguez H. (2013). Thigh compartment syndrome in urban trauma: bullets to blame, not collisions. J. Surg. Res..

[3] Ojike N.I., Roberts C.S., Giannoudis P.V. (2010). Compartment syndrome of the thigh: a systematic review. Injury.

[4] Purnell J., Monument M.J., Johnston K. (2020). Proximal hamstring tendon avulsion resulting in acute posterior thigh compartment syndrome: a case report. JBJS Case Connector.

[5] De Labachelerie C., Laboute E., Faure P. (2020). Rééducation postopératoire après réinsertion d’une lésion proximale des ischio-jambiers: à propos d’un cas avec une atteinte bilatérale et revue de la littérature. Journal de Traumatologie du Sport.

[6] Golan E.J., Bradley J.P. (2019). Proximal hamstring tears and syndrome. Oper. Tech. Orthop..

[7] Sonnery-Cottet B., Archbold P., Thaunat M. (2012). Proximal hamstring avulsion in a professional soccer player. Orthop. Traumatol. Surg. Res..

[8] Fletcher A.N., Cheah J.W., Nho S.J. (2021). Proximal hamstring injuries. Clin. Sports Med..

[9] Rodriguez J., Suneja N., Von Keudell A. (2022). Surgical demographics of acute thigh compartment syndrome. Injury.

[10] Slade H.J.T., De Ridder K. (2020). A rare cause of acute compartment syndrome in the thigh: a case report. Journal of Surgica.

[11] Primavera M, Gatti A, Tarantino U, et al. Posterior thigh compartment syndrome associated with hamstring avulsion and antiplatelet therapy. Acta Biomedica Atenei Parmensis2020; 91: 111. 10.23750/abm.v91i4.9185 PMC7927490 33525301

[12] Rameder P., Schmidt R., Machold W. (2019). Epidemiology, treatment and outcome after compartment syndrome of the thigh in 69 cases – experiences from a level I trauma centre. Injury.

[13] Fouasson-Chailloux A., Menu P., Dauty M. (2020).

[14] Sohrabi C., Mathew G., Maria N., Kerwan A., Franchi T., Agha R.A. (2023). The SCARE 2023 guideline: updating consensus Surgical CAse REport (SCARE) guidelines. Int J Surg Lond Engl..

